# The Law Relating to Infectious Diseases.—VI

**Published:** 1902-09-27

**Authors:** J. E. R. Stephens

**Affiliations:** Barrister-at-Law, Author of "Digest of Public Health Cases," etc. etc.


### The Institutional Workshop.

### THE LAW RELATING TO INFECTIOUS DISEASES.—VI.

By J. E. R. Stephens, Barrister-at-law, Author of "Digest of Public Health Cases," etc. etc.

### Hospitals.

By section 131 of the Public Health Act, 1875, any local authority may provide for the use of the inhabitants of their district, hospitals or temporary places for the reception of the sick, and for that purpose may themselves build such hospitals or places of reception, or contract for the use of any such hospital or part of a hospital, or place of reception, or enter into any agreement with any person having the management of any hospital for the reception of the sick inhabitants of their district, on payment of such annual or other sums as may be agreed on. Two or more local authorities may combine in providing a common hospital.

If it appear to the guardians of any union desirable that any hospital or building vested in them as guardians under the Acts relating to the relief of the poor, should be vested in them, as the rural sanitary authority of such union, for the reception of persons suffering from any dangerous infectious disorder, the guardians may by resolution to be confirmed by an order of the Local Government Board, transfer such hospital or building accordingly; and from and after the date named in the order, such hospital or building shall be deemed to be vested in the guardians as the rural sanitary authority of the union, for the use of the inhabitants of the union, or part thereof named in the resolution and order. (42 and 43 Vict. c. 54, s. 14.)

Where the Infectious Disease (Prevention) Act, 1890, has been adopted, the local authority are required to provide, free of charge, temporary shelter or house accommodation, with any necessary attendants for the members of any family in which infectious disease has appeared, who have been compelled to leave their dwellings to allow them to be disinfected by the local authority.

The Epidemic (and other Diseases Prevention) Act, 1883, enacts that when the Local Government Board have made regulations for any of the purposes in sect. 131 of the Act of 1875, those purposes shall be deemed to be purposes for which the sanitary authority may borrow money, as if they were "works" for which loans may be granted under the Act of 1875, and that such loans may be made forthwith and without any preliminary public notice or inquiry, if it appears to the Local Government Board desirable in order to the prompt and effective execution of such regulations.

By sect. 132 of the Public Health Act, 1875, any expenses incurred by a local authority in maintaining in a hospital or in a temporary place for the reception of the sick (whether or not belonging to such authority) a patient who is not a pauper, shall be deemed to be a debt due from such patient to the local authority, and may be recovered from him at any time within six months after his discharge from such hospital or place of reception, or from his estate in the event of his dying in such hospital or place.

In places where the Infectious Disease (Prevention) Act, 1890, has been adopted any Justice of the Peace acting in and for the district of the local authority, upon proper cause shown to him, may make an order directing the detention in hospital at the cost of the local authority of any person suffering from any infectious disease, who is then in an hospital for infectious disease, and would not on leaving such hospital be provided with lodging or accommodation in which proper precautions could be taken to prevent the spreading of the disorder by such person. Any order so to be made by any such Justice may be limited to some specific time, but with full power to any Justice to enlarge such time as often as may appear to him to be necessary. It shall be lawful for any officer of the local authority or inspector of police acting in the district, or for any officer of the hospital, on any such order being made, to take all necessary measures and do all necessary acts for enforcing the execution thereof (sect. 4).

Persons suffering from infectious disease and being without proper lodging or accommodation, may be removed to a hospital under a justice's order (P. H. A. 1875, sect. 124). Any local authority may, with the sanction of the Local Government Board, themselves provide or contract with any person to provide a temporary supply of medicine and medical assistance for the poorer inhabitants of their district.

Of late years the importance of having infectious hospitals in readiness to isolate the early cases of an epidemic has been recognised to a greater extent than it formerly was; but unfortunately many districts are still without them, and many sanitary authorities are indisposed to provide them. With a view to promote their establishment, the Isolation Hospitals Act, 1893, was passed. This Act does not apply to London or to any county borough, nor to any borough with a population of 10,000 or upwards, according to the census for the time being, without the consent of the council of the borough; nor to any borough with a smaller population without the like consent, unless the Local Government Board by order direct that the Act shall apply to it.

Subject to these limitations, the Act enables every county council to provide an isolation hospital, *i.e.*, an hospital for the reception of patients suffering from infectious disease, or to cause one to be provided in any district in their county, on application being made to them in manner provided by the Act, and on satisfactory proof being adduced that such a hospital is required. The application may be made by any urban or rural sanitary authority, or as regards any rural parish, by the vestry (after the appointed day by the parish council or parish meeting), or by any number of ratepayers not less than 25. It must be made by petition, and must state the district for which it is alleged that the hospital is required, and the reasons which the petitioners adduce for its establishment. Any such petitions must be considered by the county council, by themselves or by a committee of their body appointed for that purpose; and if they are satisfied that a *primâ facie* case is made out, they must cause a local inquiry to be held into the necessity for the establishment of an isolation hospital, the proper site for it, and the district for which it is to be established. The inquiry will be held by a committee consisting of such number of the members of the council, either with or without the addition of such other persons, or in such other manner, as the council think expedient. Due notice of the time and place at which it is to be held must be given, and any persons interested may attend and state their case.

Without any such application being made to them, the county council may, on their own initiative, direct an inquiry to be made by the medical officer of health of the county as to the necessity of an isolation hospital being established for the use of the inhabitants of any particular district in the county; and in the event of his reporting that such a hospital ought to be established, they may take the same proceedings in all respects for its establishment as if a petition had been presented by the local authority for the district named in his report.

On conclusion of their local inquiry as to the necessity for the establishment of an isolation hospital, the county council must make an order, either dismissing the petition, or constituting a hospital district, and directing an isolation hospital to be established for it.

Every person admitted into the hospital may be charged such sum as the hospital committee may think sufficient to defray the "patient's expenses" incurred in respect of him; and there may be added thereto in the case of persons brought from beyond the hospital district such sum as the committee may think fit, as a contribution to the structural and establishment expenses. Persons desirous of being provided with accommodation of an exceptional character may be so provided on their undertaking, to the satisfaction of the committee, to pay for the same a sum fixed by the committee, and also pay for all other expenses incurred in respect of their maintenance in the hospital. Patients' expenses, in respect of any person who at the time of his reception into the hospital, or at any time within 14 days previously, is or has been in receipt of Poor-law relief, will be a debt due to the hospital committee from the guardians of the union from which he is sent, and will be recoverable from them in a summary manner or otherwise. In respect of a non-pauper patient they will be a debt due to the hospital committee, and recoverable in a summary manner from the local authority of the local area from which the patient is sent. Special patients' expenses will be a debt recoverable in a summary manner from the patient, or from the estate of the patient, in respect of whom the expenses have been incurred. The expenses of the burial of any patient dying in the hospital will be payable in the same manner in which the expenses of his maintenance are payable.

A county council may, where they deem it expedient so to do for the benefit of the county, contribute out of the county rate a capital or annual sum towards the structural and the establishment expenses of an isolation hospital under the Act, or to either class of such expenses. A county council may borrow on the security of the county rates and in manner provided by the Local Government Act, 1888, any money required for the purpose of carrying into effect the provisions of this Act; and any loans so borrowed, and any other money expended by them for the purposes of the Act, together with interest thereon at the rate of four pounds per centum per annum, will be repaid to them out of the local rate. A person will not by reason of his being admitted into and maintained in a hospital established in pursuance of the Act suffer any disqualification or any loss of franchise or other right or privilege.

